# Acute macular neuroretinopathy following COVID-19 vaccination

**DOI:** 10.1038/s41433-021-01610-1

**Published:** 2021-06-22

**Authors:** Anders Djupesland Bøhler, Marianne Ekornes Strøm, Kjell Ulrik Sandvig, Morten Carstens Moe, Øystein Kalsnes Jørstad

**Affiliations:** 1 Department of Ophthalmology, Oslo University Hospital, Oslo, Norway; 2 Faculty of Medicine, University of Oslo, Oslo, Norway; 3 Oslo Eye Centre, Oslo, Norway

**Keywords:** Viral infection, Retinal diseases, Vision disorders, Adverse effects

The complex immunological mechanisms of vaccines bring about an inevitable risk of immune-mediated adverse reactions. Of special interest in this time of epidemic is the safety of COVID-19 vaccines and, in particular, the emerging evidence that the ChAdOx1 nCoV-19 adenoviral vector vaccine from AstraZeneca can cause vaccine-induced immune thrombotic thrombocytopenia (VITT). We present the case of a patient who developed an acute paracentral scotoma after having received this vaccine.

A 27-year-old female was referred for ophthalmology evaluation because of visual disturbances in her left eye. Her past medical history was unremarkable. Her only medication was an oral contraceptive (combined desogestrel and ethinylestradiol). As she worked in a hospital, she had been prioritised for COVID-19 vaccination and had recently received the first dose of the AstraZeneca vaccine. The same day she developed flu-like symptoms. These resolved 2 days later. However, a left paracentral scotoma appeared at this point.

**Fig. 1:**
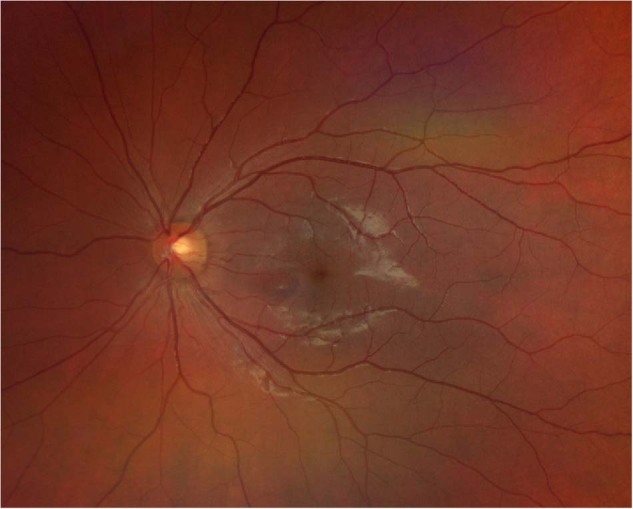
Fundus photography of the left macula. The image displays a delicate teardrop-shaped lesion nasally to the fovea.

**Fig. 2:**
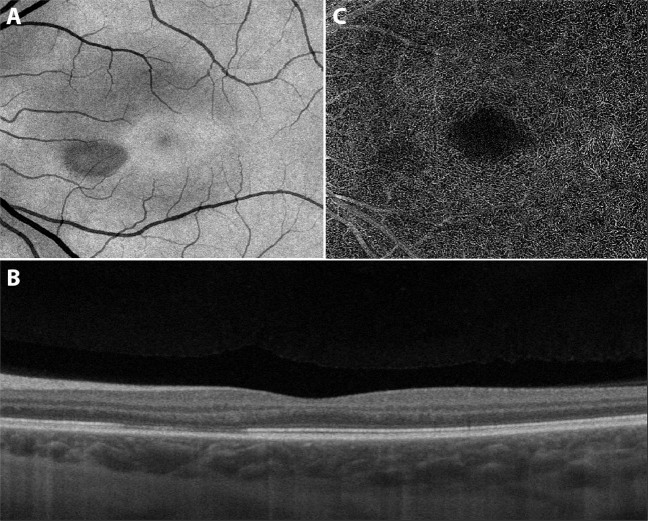
Swept source optical coherence tomography of the left macula. **A** The en face image displays a teardrop-shaped macular lesion nasally to the fovea. **B** The cross-sectional image displays slight hyperreflectivity of the outer nuclear and plexiform layers and disruption of the ellipsoid zone corresponding to the lesion. **C** The angiogram indicates subtle dropout in the deep capillary plexus corresponding to the lesion.

On clinical examination the best-corrected visual acuity was 20/20 in both eyes. Threshold perimetry of the left eye showed a modest paracentral scotoma in the upper temporal quadrant. The intraocular pressure was normal in both eyes, and there were no signs of intraocular inflammation. Fundoscopy of the left eye revealed a delicate teardrop-shaped macular lesion nasally to the fovea (Fig. 1), which was visualised better on swept source optical coherence tomography (SS-OCT) en face images (Fig. 2A). Cross-sectional SS-OCT of the lesion demonstrated slight hyperreflectivity of the outer nuclear and plexiform layers and disruption of the ellipsoid zone (Fig. 2B). SS-OCT angiography indicated subtle dropout in the deep capillary plexus corresponding to the lesion (Fig. 2C). There was no evidence of VITT; laboratory work-up showed a normal complete blood count, negative C-reactive protein and absence of antibodies to platelet factor 4-polyanion complexes.

The signs and symptoms of our patient were consistent with acute macular neuroretinopathy (AMN). The exact pathophysiology of AMN is unknown, but several risk factors have been identified; the most common associations are non-specific flu-like illness or fever and use of oral contraceptives [1]. The present case further expands the spectrum of possible associations. To the best of our knowledge, AMN following COVID-19 vaccination has not been previously published, and a coincidental finding cannot be ruled out. However, there are case reports of AMN in the course of COVID-19 infection [2–4]. An association between AMN and both COVID-19 infection and vaccination raises the question as to whether a common immune-mediated pathway can trigger this peculiar macular disease.
